# C-type lectins and phagocytosis

**DOI:** 10.1016/j.imbio.2008.11.003

**Published:** 2009-07

**Authors:** Ann M. Kerrigan, Gordon D. Brown

**Affiliations:** Institute of Infectious Disease and Molecular Medicine, CLS, Faculty of Health Sciences, University of Cape Town, Observatory, Cape Town 7925, South Africa

**Keywords:** Collectin, DC-SIGN, Dectin-1, Mannose receptor, Pattern recognition receptor, COPD, chronic obstructive pulmonary disorder, CTLD, C-type lectin-like domain, CR, complement receptor, DC, dendritic cell, DC-SIGN, DC specific ICAM grabbing non-integrin, FcγR, Fcγ receptor, IgG, immunoglobulin G, ICAM, intercellular adhesion molecule, ITAM, immunoreceptor tyrosine-based activation motif, LPS, lipopolysaccharide, MASP, MBL-associated serine protease, MBL, mannose binding lectin, MR, mannose receptor, PAMP, pathogen associated molecular pattern, PRR, pattern recognition receptor, PTX, pentraxin, SP, surfactant protein, TLR, toll-like receptor

## Abstract

To recognise and respond to pathogens, germ-line encoded pattern recognition receptors (PRRs) bind to conserved microbial structures and activate host defence systems, including microbial uptake by phagocytosis. Phagocytosis is a complex process that is instrumental in the control of extracellular pathogens, and this activity is mediated by several PRRs, including a number of C-type lectins. While some of these receptors have clearly been shown to mediate or regulate the uptake of pathogens, others are more contentious and are less well understood in terms of their phagocytic potential. Furthermore, very little is known about the underlying phagocytic mechanisms. Here, we review the phagocytic roles of the mannose receptor, Dectin-1, dendritic cell-specific ICAM grabbing non-integrin (DC-SIGN), DCL-1, mannose binding lectin and surfactant proteins A and D.

## Introduction

Phagocytosis is an actin-dependent mechanism by which cells (phagocytes) ingest large particles that are usually greater than 0.5μm in diameter (Aderem and Underhill, 1999). Phagocytosis is phylogenetically conserved in mammals and has evolved into a remarkably intricate process. Elie Metchnikoff was the first to describe it in the late nineteenth century and he was awarded the Nobel Prize in Physiology or Medicine in 1908 for his discovery. One hundred years later, Metchnikoff's cellular theory of immunity has stood the test of time, but we are now aware that the phagocytic process is much more complex than originally envisioned and we are only just beginning to decipher its various mechanistic and molecular workings.

**Fig. 1:**
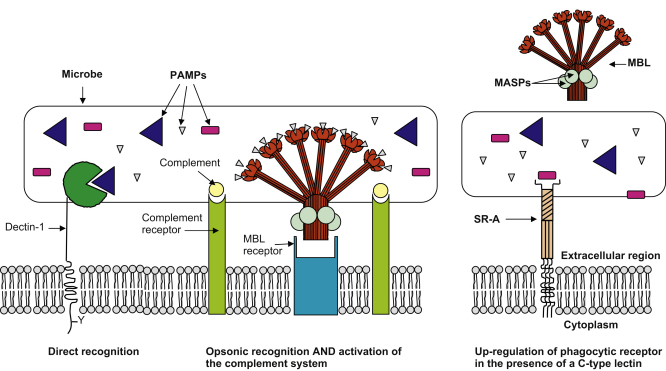
Direct recognition, opsonisation, complement activation and receptor up-regulation by C-type lectins. Phagocytic C-type lectins can directly recognise PAMPs on the surface of microbes and mediate phagocytosis (e.g. Dectin-1). Alternatively, soluble C-type lectins can interact directly with pathogens to promote opsonisation of the microbe (e.g. MBL) which can subsequently be phagocytosed via specific receptors. In addition, some C-type lectins can activate complement leading to its deposition on the microbial surface and phagocytosis mediated by complement receptors (e.g. MBL associated MASPS are activated on binding to pathogens which in turn cleave complement components and activate the complement system). Finally, C-type lectins can cause up-regulation of other phagocytic receptors, independently of their binding to the microbe (e.g. MBL up-regulation of SR-A).

Phagocytic cells are involved in a number of biological processes, including the recognition and control of invading microbes. Innate pathogen recognition is mediated by a series of germ-line encoded pattern recognition receptors (PRRs) that are either soluble or membrane-bound. These PRRs recognise conserved microbial structures, such as bacterial lipopolysaccharide or fungal β-glucans, that are known as pathogen associated molecular patterns (PAMPs) (Janeway and Medzhitov, 2002). Soluble PRRs include the collectins, ficolins, pentraxins and complement. These proteins coat or “opsonise” the infectious agent which can then be ingested via specific opsonic receptors. Some of these proteins can also regulate the surface expression of other phagocytic receptors and thereby exert an indirect influence on uptake. Membrane-bound PRRs, such as Dectin-1, directly recognise microbes and mediate their uptake (Fig. 1).

Although not discussed in detail here, the role of toll-like receptors (TLRs) in phagocytosis is a topic which is fervently debated in the literature. One side of this debate argues that signalling through surface TLRs, which are recruited to the phagosome upon uptake of microbial pathogens, is critical for phagosome maturation (Blander, 2007a, b; Blander and Medzhitov, 2004, 2006a, b). The other side argues that phagosome maturation proceeds independently of TLR signalling (Russell and Yates, 2007; Yates et al., 2005; Yates and Russell, 2005). TLR signalling has also been shown to participate in autophagy (Xu et al., 2007) and a recent study reports that engagement of the autophagy pathway via TLR signalling enhances phagosome maturation (Sanjuan et al., 2007). This makes it tempting to assign a role for TLR signalling in the regulation of phagosome maturation, but further studies are first needed to clarify the current ambiguities.

**Fig. 2:**
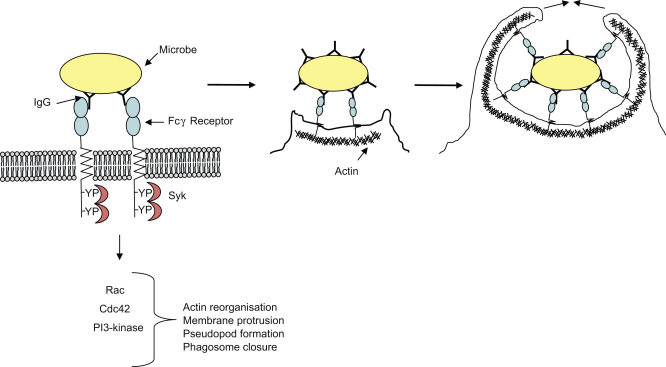
Schematic representation of FcγR signalling leading to recruitment of Syk, activation of Rac, Cdc42 and PI3-kinase, and engulfment of microbe via “zippered” phagocytosis.

The FcγRs and complement receptor 3 (CR3) are involved in the uptake of opsonised pathogens and are the two best characterised phagocytic receptors in macrophages (for extensive reviews see Aderem and Underhill, 1999; Garcia-Garcia and Rosales, 2002; Swanson and Hoppe, 2004). FcγRs bind to immunoglobulin G (IgG)-opsonised particles, whereas CR3 binds complement-coated particles. The reorganisation of actin underlies uptake by both receptors but the mechanisms are distinct. It has long been known that during FcγR-mediated phagocytosis (Fig. 2), actin-rich pseudopodia extend circumferentially around the particle and draw it into the cell forming a tight-fitting “zippered” phagosome (Griffin et al., 1975; Kaplan, 1977). In contrast, complement-opsonised particles appear to sink into the cell with little or no protrusions resulting in a more spacious phagosome (Griffin et al., 1975; Kaplan, 1977). During FcγR-mediated phagocytosis, which is mediated by signalling through defined cytoplasmic immunoreceptor tyrosine-based activation (ITAM) motifs, PI-3 kinase, Rac and Cdc42 have been shown to have essential roles in actin reorganisation, membrane protrusion, pseudopod extension and phagosome closure (Caron and Hall, 1998). On the other hand, Rho is required for CR3-mediated phagocytosis, whereas tyrosine kinases, Cdc42 and Rac are not critical (Caron and Hall, 1998). In addition, unlike FcγR-mediated phagocytosis, induction of the respiratory burst and production of inflammatory mediators do not accompany CR3-mediated phagocytosis (Aderem et al., 1985; Stein and Gordon, 1991; Wright and Silverstein, 1983; Yamamoto and Johnston, 1984). In contrast, relatively little is known about mechanisms underlying C-type lectin-mediated phagocytosis and indeed the precise role of certain C-type lectins in phagocytosis is still contentious.

## C-type lectins and phagocytosis

**Fig. 3:**
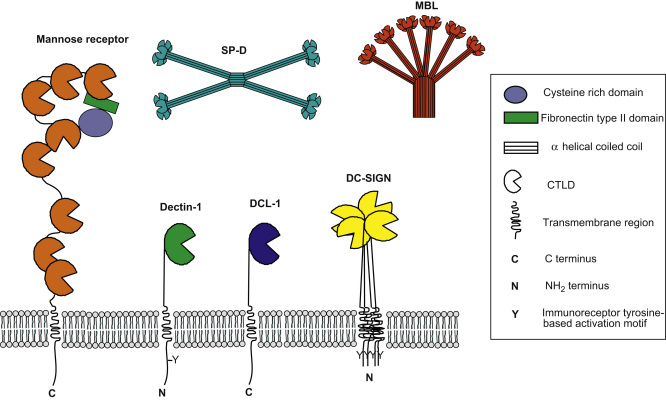
Structural representation of C-type lectin proteins. Mannose receptor (“bent” conformation), Dectin-1, DCL-1 and oligomers of DC-SIGN, mannose binding lectin and surfactant protein-D are shown. Not drawn to scale.

C-type lectins were originally understood to be ${\mathrm{Ca}}^{2+}$-dependent carbohydrate binding proteins containing a conserved carbohydrate recognition domain. It has since been determined that other proteins contain the same domain, yet do not necessarily bind carbohydrates or ${\mathrm{Ca}}^{2+}$. The C-type lectin superfamily is a large group of proteins that are characterised by the presence of one or more C-type lectin-like domains (CTLDs). The superfamily is divided into 17 groups based on their phylogeny and domain organisation (Drickamer and Fadden, 2002; Zelensky and Gready, 2005). Despite the presence of a highly conserved domain, C-type lectins are functionally diverse and have been implicated in various processes including cell adhesion, tissue integration and remodelling, platelet activation, complement activation, pathogen recognition, endocytosis, and phagocytosis. Here, we will review the phagocytic potential of C-type lectins (Table 1, Fig. 3).

## The mannose receptor (MR)

The mannose receptor (MR) (CD206) is a type-I transmembrane protein that is characterised as a Group VI C-type lectin. It shares the same overall structure with three other receptors (phospholipase A2 receptor, ENDO 180 and DEC205) which together are known as the MR family (East and Isacke, 2002). Structurally, it consists of an extracellular region containing an amino terminal cysteine rich domain, a fibronectin type II repeat domain, eight CTLDs, a transmembrane region and a short cytoplasmic tail. A structural model has been proposed where at least two conformations of the MR exist – an extended form and a more compact “bent” form (Boskovic et al., 2006) (Fig. 3). The MR has a single tyrosine residue in its cytoplasmic tail that occurs within a diaromatic amino acid sequence (Kruskal et al., 1992). Its expression was originally thought to be restricted to mammalian tissue macrophages but it is now known to be expressed on lymphatic and hepatic epithelium, kidney mesangial cells, tracheal smooth muscle cells and retinal pigment epithelium (Lew et al., 1994; Linehan et al., 1999; Shepherd et al., 1991). Expression has also been observed on human monocyte-derived DCs (Avrameas et al., 1996; Engering et al., 1997; Sallusto et al., 1995) and on a subpopulation of murine DCs (McKenzie et al., 2007). The bulk of the MR is intracellular, located within the endocytic pathway, with only a small proportion present on the cell surface. Its expression is up-regulated by IL-4, IL-13 and IL-10, whereas IFNγ has a down-regulatory effect (Doyle et al., 1994; Harris et al., 1992; Martinez-Pomares et al., 2003; Stein et al., 1992). Surface expression is also influenced by proteolytic cleavage of the membrane-bound receptor by a metalloprotease resulting in a functional soluble form of the receptor (Martinez-Pomares et al., 1998).

The MR binds a broad array of microorganisms, including *Candida albicans*, *Pneumocystis carinii*, *Leishmania donovani*, *Mycobacterium tuberculosis*, and capsular polysaccharides of *Klebisella pneumoniae* and *Streptococcus pneumonia* (Chakraborty et al., 2001; Ezekowitz et al., 1991; Marodi et al., 1991; O’Riordan et al., 1995; Schlesinger, 1993; Zamze et al., 2002). The receptor recognises mannose, fucose or N-acetylglucosamine sugar residues on the surfaces of these microorganisms (Largent et al., 1984) and carbohydrate recognition is mediated by CTLDs 4–8 (Taylor et al., 1992). The MR has been implicated in the phagocytic uptake of pathogens, but there are limited examples actually demonstrating MR-dependent phagocytosis. The first suggestion that the MR was a phagocytic receptor was based on the mannan-inhibitable uptake of zymosan by mouse peritoneal macrophages (Sung et al., 1983). It has subsequently been shown that mannan can be recognised by a number of receptors, including DC specific ICAM grabbing non-integrin (DC-SIGN), and studies that have attributed a phagocytic role to the MR based purely on experiments using mannan as a specific inhibitor of the MR are therefore not reliable. Others have reported that transfection of the non-phagocytic COS-1 cell line with the MR results in phagocytosis of *C. albicans* and *P. carinii* and that the cytoplasmic tail of the receptor is essential for this activity (Ezekowitz et al., 1990, 1991). A further study showed that MR-positive J774-E macrophages ingested threefold more *Francisella tularensis* than MR-negative J744-E cells. This study used receptor-blocking antibody in addition to soluble mannan as inhibitors (Schulert and Allen, 2006). In macrophages the MR is also thought to be involved in the nonopsonic phagocytosis of virulent *M*. *tuberculosis* (Schlesinger, 1993). MR recognition of mannose-capped lipoarabinomannan (ManLAM) on the mycobacterial cell wall initiates a specific phagocytic pathway resulting in limited phagosome–lysosome fusion (Kang et al., 2005), suggesting a mechanism of how the pathogen survives in the phagosome.

The MR has also been implicated in the phagocytic uptake of apoptotic cells in COPD (Hodge et al., 2003). Alveolar macrophages from COPD patients express significantly less MR than alveolar macrophages from healthy controls. This link was more firmly established when the phagocytic ability of alveolar macrophages was shown to be significantly reduced by blocking the expression of the MR using a specific blocking antibody (Hodge et al., 2008).

There has been some examination of the mechanism of MR-mediated phagocytosis. The cytoplasmic tail is required for uptake in MR-transfected cells, however, mutation of the single cytoplasmic tyrosine reduced, but did not abolish phagocytosis (Kruskal et al., 1992). F-actin, talin, PKCα, MARCKS and Myosin I are recruited to the phagosome, but in contrast to phagocytosis by FcγR and CR3, vinculin and paxillin are not recruited during MR-mediated phagocytosis (Allen and Aderem, 1996). Further studies in macrophages suggest that the mechanism may require focal F-actin polymerisation, Cdc42 and Rho activation, promoting PAK1 activation and requiring the Rho effector molecule ROCK, but not Rac (Zhang et al., 2005). It has also been suggested that non-opsonised zymosan is internalised largely through the MR in a PI-3K p110α-dependent manner (Lee et al., 2007). However, the lack of defined cytoplasmic signalling motifs makes it difficult to understand how signalling occurs. Indeed, the ability of the MR to mediate phagocytosis has recently been challenged, as a number of different cell lines expressing the MR failed to show internalisation of known ligands (Le Cabec et al., 2005). Le Cabec et al. propose that the MR is not a professional phagocytic receptor but may require interaction with other ‘partners’ present in macrophages and dendritic cells to mediate particle uptake (Le Cabec et al., 2005). However, there are still reports emerging regarding the involvement of the MR in phagocytosis. Very recently it has been shown that the MR is found around *C*. *albicans* containing phagosomes in human DCs (Cambi et al., 2008). Evidently there are contradictions in the literature and further research is required to clarify whether the MR is a professional phagocytic receptor or a more minor player in the process.

## Dectin-1

Dectin-1 is a type II transmembrane protein that is classified as a Group V non-classical C-type lectin and lacks the conserved residues involved in the ligation of calcium that are usually required to co-ordinate carbohydrate binding. It consists of a single extracellular CTLD connected by a stalk to a single transmembrane region, and a cytoplasmic tail which contains an ITAM-like motif (Fig. 3). There are two major isoforms of Dectin-1, one encoding the full length receptor and a version which lacks the stalk (Heinsbroek et al., 2006). Murine Dectin-1 is widely expressed by many cell types, including macrophages, dendritic cells, monocytes, neutrophils and a subset of splenic T cells (Taylor et al., 2002). The expression of human Dectin-1 differs slightly in that it is also expressed on B cells, eosinophils and mast cells (Olynych et al., 2006; Willment et al., 2005). Studies on murine Dectin-1 expression have shown that it can be influenced by various cytokines and microbial factors. For example IL-4, IL-13 and GM-CSF cause Dectin-1 expression to be highly up-regulated. In contrast IL-10, LPS and dexamethasone cause down-regulation of Dectin-1 expression (Willment et al., 2003).

Dectin-1 was initially identified as a dendritic cell specific receptor that modulates T cell function through recognition of an unidentified ligand (Ariizumi et al., 2000; Grunebach et al., 2002). It was subsequently reidentified as a receptor for β-glucans, which are carbohydrate polymers found primarily in the cell walls of fungi, but also in plants and some bacteria (Brown and Gordon, 2001, 2003). The ${\mathrm{Ca}}^{2+}$ independent mechanism for recognition of carbohydrates by Dectin-1 is still unclear, however, it is known that at least two residues flanking a shallow groove on the protein surface, Trp221 and His223, are crucial for β-glucan binding (Adachi et al., 2004). By way of its β-glucan specificity, Dectin-1 can recognise a number of fungal species, including *C. albicans, P. carinii, Saccharomyces cerevisiae, Coccidioides posadasii* and *Aspergillus fumigatus* (Brown et al., 2003; Gersuk et al., 2006; Saijo et al., 2007; Steele et al., 2003, 2005; Taylor et al., 2007; Viriyakosol et al., 2005).

The presence of an ITAM-like motif in the cytoplasmic tail of Dectin-1 initially suggested similarity to FcγRs. During FcγR-mediated internalisation, src kinases phosphorylate the tyrosine residues within the ITAM leading to recruitment and activation of Syk kinase. Syk activation then initiates a flood of signalling events that ultimately result in various cellular responses, including phagocytosis (Garcia-Garcia and Rosales, 2002). PI-3 kinase is required for pseudopod extension and phagosomal closure during FcγR-mediated uptake, as well as the previously mentioned Rho and Cdc42 GTPases, which are required for actin reorganisation (Fig. 2).

The ligation of Dectin-1 also triggers intracellular signalling resulting in a variety of cellular responses, including phagocytosis. In contrast to uptake by FcγR, however, phagocytosis by Dectin-1 requires phosphorylation of only the membrane proximal tyrosine of the ITAM-like motif (Herre et al., 2004). Src kinases are only partially responsible for this, as the inclusion of PP2, a src kinase inhibitor, in internalisation experiments, did not cause complete inhibition of uptake. In addition, Syk was not required for Dectin-1 mediated phagocytosis in macrophages, indicating the existence of novel signalling pathway(s) (Herre et al., 2004). On the other hand, Syk is required, at least in part, for Dectin-1 dependent uptake in dendritic cells and in NIH-3T3 cells, suggesting that signalling events initiated from Dectin-1 may differ depending on cell type (Herre et al., 2004; Rogers et al., 2005). Dectin-1 also has a highly charged triacidic motif (DED) in the ITAM-like motif which is required for uptake (Underhill et al., 2005). Inhibition studies have shown that PI-3 kinase was not essential for Dectin-1 mediated phagocytosis, whereas PKC was required. In addition, the use of dominant-negative constructs have shown that Cdc42 and Rac-1 are involved, but not Rho (Herre et al., 2004; Ueyama et al., 2004). The route of intracellular processing of Dectin-1 may depend on the molecular nature of the ligand, with the receptor directed to lysosomes during uptake of the particulate ligand zymosan, but recycled to the membrane during uptake of the soluble ligand, laminarin (Herre et al., 2004).

Other molecules may also associate with Dectin-1 during phagocytosis. For example, immunoprecipitation experiments have shown that the tetraspanin, CD63 associates with Dectin-1 (Mantegazza et al., 2004). In addition, phagocytosis of yeast particles by DCs was accompanied by a decrease in CD63 expression, which was inhibitable by laminarin. Although the functional significance of a Dectin-1-CD63 interaction has not yet been elucidated, it may represent part of a signalling complex that could influence phagocytosis (Brown, 2006). Dectin-1 also recognises opsonised particles. Pentraxin-3 (PTX-3) causes aggregation of zymosan, leading to the Dectin-1 dependent internalisation of an increased number of zymosan particles (Diniz et al., 2004). Moreover, macrophages express higher levels of PTX-3 in the presence of zymosan and it has been suggested that during fungal infection, secreted PTX-3 may enhance clearance by opsonising the pathogen and facilitating its uptake by Dectin-1 (Diniz et al., 2004).

## DC-SIGN (CD209)

DC-SIGN is a type II transmembrane protein that is classified as a Group II C-type lectin. It consists of an extracellular C-terminal CTLD, a repetitive stalk region which mediates receptor multimerisation, a single transmembrane region and a cytoplasmic tail within which a number of internalisation motifs are present (Fig. 3). Its expression was initially believed to be restricted to immature dendritic cells, but it is now known to be expressed on endothelium and selected macrophage subpopulations (Geijtenbeek et al., 2000b; Krutzik et al., 2005; Lai et al., 2006; Soilleux et al., 2002; Tailleux et al., 2005). DC-SIGN expression is mainly induced by IL-4, and is negatively regulated by IFNγ, TGFβ, and dexamethasone (Relloso et al., 2002).

DC-SIGN was originally identified as a receptor for intercellular adhesion molecule-3 (ICAM-3) that facilitates DC-mediated T-cell proliferation and binds HIV-1 (Geijtenbeek et al., 2000a, b). It has since been reported that the receptor interacts with a range of pathogens, including *M. tuberculosis, C. albicans, Helicobacter pylori, Schistosoma mansoni* and *A. fumigatus* ( Appelmelk et al., 2003; Cambi et al., 2008; Geijtenbeek et al., 2000b, 2003; Serrano-Gomez et al., 2004; Tailleux et al., 2003; van Die et al., 2003). DC-SIGN recognises both internal mannose branched structures and terminal di-mannoses, and the receptor forms tetramers aiding its specificity for high mannose oligosaccharides (Mitchell et al., 2001).

DC-SIGN is often described as a phagocytic receptor. This is conceivable given its interactions with pathogens and the presence of internalisation motifs (di-leucine motif, tri-acidic cluster, ITAM motif) in its cytoplasmic tail (Zhou et al., 2006). However, to date, the evidence for the phagocytic potential of DC-SIGN has been indirect and still needs to be demonstrated conclusively. For example, a study by Serrano-Gomez et al. shows that DC-SIGN mediates efficient binding of *A. fumigatus* conidia to immature and mature MDDC (Serrano-Gomez et al., 2004). In this study, internalisation assays in immature MDDC resulted in conidial uptake in vesicles that stained positively for DC-SIGN indicating a potential role for the receptor in uptake. It has also been shown that DC-SIGN is present in *M. tuberculosis* vacuoles of MDDC (Tailleux et al., 2003). Other studies have similarly shown colocalisation of DC-SIGN with phagosomes (Cambi et al., 2008; Geijtenbeek et al., 2003). The demonstration of phagocytosis by usually non-phagocytic cells transduced with DC-SIGN would be a more direct way to examine the phagocytic potential of the receptor. This type of approach has been used by Zhang et al. where they indirectly demonstrated internalisation of a non-pathogenic strain of *Escherichia coli* by HeLa cells transfected with DC-SIGN (Zhang et al., 2006). Interestingly, mutation of the tyrosine in the cytoplasmic domain of DC-SIGN did not affect internalisation of *E. coli* in these assays. In a subsequent study, they used the DC-SIGN transfected HeLa cells to demonstrate uptake of *Yserina pestis* (Zhang et al., 2008). These studies go some way to implicating DC-SIGN as a phagocytic receptor, however, further work in alternative cell lines is warranted, as *Y. pestis* also binds to heparan sulphate proteoglycan receptor on HeLa cells, which contributes to its internalisation.

There have been no reports of a mechanism for DC-SIGN mediated phagocytosis. However, activation of DC-SIGN triggers Rho-GTPase (Hodges et al., 2007) making it conceivable that Rho could be involved in phagocytosis mediated by this receptor. Further studies are required to confirm the phagocytic ability of DC-SIGN and elucidate the underlying mechanism(s).

## DCL-1 (CD302)

DCL-1 is a recently described type I transmembrane protein which was identified as a genetic fusion partner of human DEC-205 in Hodgkin's lymphoma cell lines and is classified as a Group XV C-type lectin (Kato et al., 2003; Zelensky and Gready, 2005). The name DCL-1 is derived from DEC-205-associated C-type lectin-1 and the receptor consists of a single extracellular CTLD, a short spacer followed by a transmembrane region and a cytoplasmic tail containing a putative tyrosine-based internalisation motif (Fig. 3). Human DCL-1 (hDCL-1) is expressed on monocytes, macrophages, granulocytes and dendritic cells (Kato et al., 2007). The receptor does not contain the amino acids that coordinate ${\mathrm{Ca}}^{2+}$-dependent sugar binding in other C-type lectins, suggesting that it does not have classic sugar binding capacity, but no endogenous or exogenous ligands have yet been identified. A recent study has shown that cell lines transfected with hDCL-1 efficiently phagocytose microbeads coated with hDCL-1 monoclonal antibodies. However, monocyte derived macrophages displayed relatively inefficient binding and phagocytosis of these antibody-coated microbeads (Kato et al., 2007). Further *in vitro* and *in vivo* studies are needed in order to clarify the potential phagocytic role of DCL-1.

## Soluble C-type lectins

### Mannose-binding lectin (MBL)

Mannose-binding lectin (MBL) is a Group III C-type lectin belonging to the collectins (Holmskov et al., 2003), which are a group of soluble oligomeric proteins containing collagenous regions and CTLDs. MBL is secreted into the blood stream as a large multimeric complex and is primarily produced by the liver, although other sites of production, such as the intestine, have been proposed (Uemura et al., 2002). The basic functional unit of MBL is a homotrimer, with each monomer consisting of an amino-terminal cysteine rich domain, a long collagenous domain, an α-helical coiled coil and a single CTLD (Fig. 3). Oligomerisation of trimeric subunits creates a bouquet-like structure and in serum, MBL consists of oligomers ranging from dimers to hexamers. It recognises carbohydrates such as mannose, glucose, l-fucose, N-acetyl-mannosamine (ManNAc), and N-acetyl-glucosamine (GlcNAc). Oligomerisation of MBL enables high avidity binding to repetitive carbohydrate ligands, such as those present on a variety of microbial surfaces, including *E. coli*, *Klebisella aerogenes*, *Neisseria meningitides*, *Staphylococcus aureus*, *S. pneumonia*, *A. fumigatus and C. albicans* (Davies et al., 2000; Neth et al., 2000; Schelenz et al., 1995; Tabona et al., 1995; van Emmerik et al., 1994).

MBL has the capacity to modify the efficiency of uptake and the expression of other phagocytic receptors. Activation of the complement system via MBL-associated serine proteases (MASPs) (Dahl et al., 2001; Kawasaki et al., 1989; Matsushita and Fujita, 1992; Stover et al., 1999; Thiel et al., 1997), results in deposition of complement on the microbial surface that can lead to uptake via complement receptors (Fig. 1) (Kawasaki et al., 1989; Neth et al., 2002). However, inhibition of bacterial growth associated with the MBL-MASP activation of complement has also been observed, without any enhancement of phagocytosis (Ip and Lau, 2004). This indicates that the specific responses induced by MBL may be dependent on the nature of the microbial target. MBL can also influence expression of other PRRs, as demonstrated by the ability of MBL to augment the uptake of *S. aureus* through the up-regulation of scavenger receptor A (SR-A) (Ono et al., 2006) (Fig. 1).

MBL has also been proposed to function directly as an opsonin by binding to carbohydrates on pathogens and then interacting with MBL receptors on phagocytic cells, promoting microbial uptake and stimulating immune responses (Fig. 1). This was first described by Kuhlman et al. who observed that binding of MBL to *Salmonella montevideo* resulted in an MBL-dependent uptake by monocytes (Kuhlman et al., 1989). Thus MBL can interact directly with receptor(s) on the surface of monocytes and several potential MBL receptors have since been proposed, although their likelihood is still debated in the literature. Calreticulin has emerged as the main candidate (Malhotra et al., 1990), but further studies are required to confirm its interaction with MBL and its role in the phagocytosis of pathogens.

A recent study has shown that MBL modifies cytokine responses through a novel cooperation with TLR2/6 in the phagosome (Ip et al., 2008). Although the stimulation of the inflammatory response was not caused by enhanced phagocytosis, bacterial engulfment was required. This study therefore demonstrates the importance of phagocytosis in providing the appropriate cellular environment to facilitate cooperation between molecules (Ip et al., 2008).

## Surfactant proteins

Surfactant proteins A and D (SP-A, SP-D) are also collectins within the Group III C-type lectins. Their basic structure is similar to MBL and includes an amino-terminal cysteine rich domain, a collagenous domain, an α-helical coiled coil and a single CTLD. They are also assembled as trimeric subunits which form oligomers. SP-D forms dodecamers that are characterised by a cruciform shape (Fig. 3) (Crouch et al., 1994). SP-A is present mainly as octadecamers with a bouquet-like structure similar to that of MBL oligomers (Hickling et al., 1998). SP-A and SP-D are primarily synthesised in the lungs by type II alveolar and Clara cells (Lu et al., 2002). They are secreted into the alveolar space where they are the main protein constituents of pulmonary surfactant. SP-A and SP-D have been shown to interact with a wide variety of carbohydrates and glycolipids, but both receptors have common and distinct ligand recognition capacities. For example, both proteins bind to glucose and mannose, however, SP-A binds preferentially to l-fucose and N-acetylmannosamine (Haagsman et al., 1987; Haurum et al., 1993), whereas SP-D displays preferential binding to glucose, maltose and inositol (Lim et al., 1994; Persson et al., 1990). Through these carbohydrate recognition abilities, both proteins have been shown to bind to a wide range of pathogens, including *Pseudomonas aeruginosa*, *P. carinii*, *A. fumigatus*, *M. tuberculosis*, *S. pneumoniae* and *K. pneumonia* (Hartshorn et al., 1998; Khubchandani et al., 2001; Madan et al., 1997; Mariencheck et al., 1999; Ofek et al., 2001; Pasula et al., 1997; Yong et al., 2003). Unlike MBL, the surfactant proteins do not activate the complement system. However, they can function directly as opsonins by binding to microbial carbohydrates and interacting with surfactant receptors on phagocytic cells (LeVine et al., 1999; Mariencheck et al., 1999; Ofek et al., 2001). A number of candidate receptors, including calreticulin have been described and are reviewed elsewhere (Gardai et al., 2003; Kishore et al., 2006).

Surfactant proteins can also cause aggregation of pathogens, which, depending on the pathogen involved, can either enhance or inhibit microbial uptake. For example, aggregation of *S. pneumoniae* by SP-D stimulates microbial uptake (Hartshorn et al., 1998), whereas SP-D mediated aggregation of *P. carinii* inhibits phagocytosis by alvelolar macrophages (Yong et al., 2003).

Similar to MBL, the surfactant proteins can also indirectly influence phagocytosis by regulating expression of other phagocytic receptors. For example, SP-A increases the expression of scavenger receptor A resulting in an enhancement of uptake of *S. pneumoniae* (Kuronuma et al., 2004). SP-A can also enhance FcR and CR1-mediated phagocytosis through unknown mechanisms, leading to its description as an activation ligand (Tenner et al., 1989).

## Concluding remarks

We have seen that a number of C-type lectins are involved in phagoctyosis. These molecules can function as phagocytic receptors, as direct or indirect opsonins, and some can also modulate expression of other receptors (Fig. 1). In certain cases, the individual C-type lectin fulfils more than one of these roles. Although not discussed in detail here, C-type lectins can also cooperate with other molecules, as in the case of MBL and TLR2 mentioned here. In general, the mechanisms of phagocytosis by C-type lectins are not very well characterised but they are clearly complex and appear to be unique to each receptor. Out of necessity, most studies have focused on particular receptors in isolation, but we should remember that the mechanism of uptake in phagocytes will be a result of the collective contribution of the several receptors involved in recognition of the specific pathogen.

## Figures and Tables

**Table 1:** C-type lectin receptors, their ligands and defined or putative signalling motifs

C-type lectin	Selected ligand(s)	Signalling motif(s)
Mannose receptor	Mannose, fucose, N-acetylglucosamine	Putative: cytoplasmic tyrosine
Dectin-1	β-Glucan	Cytoplasmic ITAM-like motif, tri-acidic motif (DED)
DC-SIGN	Mannose, ICAM-3	$\left(\begin{array}{c} \text{Di-leucine motif}\\ \text{Tri-acidic cluster}\\ \text{ITAM motif} \end{array}\right\}\text{involved in endocytosis}$
DCL-1	Unknown	Putative: cytoplasmic tyrosine
MBL	Mannose, glucose, l-fucose, ManNAc, GlcNAc	None
SP-A	Glucose, mannose, l-fucose, N-acetylmannosamine	None
SP-D	Glucose, mannose, maltose, inositol	None
